# Nuclear cell-free DNA on the loose: an early warning signal of ischemia–reperfusion injury in kidney transplantation

**DOI:** 10.3389/fimmu.2025.1704152

**Published:** 2026-01-07

**Authors:** Gabriel Strandberg, Rebecca Trattner, Myriam Martin, Carl M. Öberg, Carl Raihle, Oleg Slivca, Shahnawaz Alam, Mårten Segelmark, Anders Christensson, Bo Nilsson, Clara Paul, Anna M. Blom, Ali-Reza Biglarnia

**Affiliations:** 1 Department of Clinical Sciences Malmö, Faculty of Medicine, Lund University, Malmö, Sweden; 2 Department of Surgery, Skånes Universitetssjukhus Malmö, Malmö, Sweden; 3 Department of Translational Medicine, Lund University, Malmö, Sweden; 4 Department of Clinical Chemistry and Pharmacology, Office for Medical Services, Malmö, Sweden; 5 Department of Clinical Sciences Lund, Faculty of Medicine, Lund University, Lund, Sweden; 6 Skånes Universitetssjukhus Lund, Lund, Sweden; 7 Department of Pathology and Laboratory Medicine, Cumming School of Medicine, University of Calgary, Calgary, AB, Canada; 8 Department of Immunology, Genetics and Pathology (IGP), Rudbeck Laboratory C5:3, Uppsala University, Uppsala, Sweden

**Keywords:** cell-free DNA, ischemia-reperfusion injury, kidney transplantation, cell injury, thromboinflammation

## Abstract

**Introduction:**

Cell-free DNA is an emerging marker of allograft injury, yet its role in the immediate phase of ischemia–reperfusion injury remains incompletely understood.

**Methods:**

In this prospective cohort of 127 kidney transplant recipients (86 deceased donors, 41 living donors), intraoperative plasma samples were collected systemically pre-implantation and from the allograft vein postreperfusion. Nuclear and mitochondrial cell-free DNA were quantified, alongside subset assessments of neutrophil extracellular trap markers and soluble C5b-9 as a marker of thromboinflammation. An in vitro necrosis model of human proximal tubular cells evaluated the concordance between cell injury and C5b-9 generation.

**Results:**

An immediate and sustained release of nuclear, but not mitochondrial, cell-free DNA was observed post-reperfusion, predominantly in deceased donor kidneys. This release corresponded with cold ischemic time and delayed graft function while showing temporal correlations with soluble C5b-9, indicating that cell-free DNA release parallels thromboinflammatory activation upon reperfusion. *In vitro*, C5b-9 generation occurred on necrotic, but not viable, tubular cells, supporting the relationship between cell injury and thromboinflammation. Neutrophil extracellular trap markers did not consistently correlate with early cell-free DNA release.

**Conclusion:**

Immediate nuclear cellfree DNA release upon reperfusion reflects intragraft injury linked to downstream thromboinflammatory activation, underscoring the impact of early ischemia–reperfusion injury.

## 1 Introduction

Ischemia–reperfusion injury (IRI) is a major contributor to both early and long-term dysfunction in kidney transplantation (KT) (1, 2). In a recent work, we demonstrated that reperfusion of ischemic kidneys promptly activates the intravascular innate immune system, encompassing the complement, coagulation, and kinin–kallikrein cascades. This concurrent activation constitutes a coordinated thromboinflammatory response, with soluble C5b-9 (sC5b-9) emerging as a representative marker due to its strong correlations with innate cascade activation markers (3). Notably, elevated sC5b-9 levels during early reperfusion were associated with delayed graft function (DGF) and reduced allograft function at 2 years, findings consistent with previous reports and highlighting its prognostic relevance in transplant outcomes (4, 5).

Circulating cell-free DNA (cfDNA) has emerged as a biomarker for allograft injury, particularly in the context of rejection (6, 7). Additionally, neutrophil extracellular traps (NETs), composed of chromatin-bound histones and cfDNA, have been implicated in IRI-mediated damage and may contribute to cfDNA release in the early post-reperfusion phase (8, 9). Although cfDNA is a well-established injury marker, its early dynamics during reperfusion and its relationship to thromboinflammation and allograft function remain unclear.

We hypothesized that early cfDNA release in allograft venous return reflects acute IRI and correlates with downstream thromboinflammatory activation and allograft dysfunction. To test this, we analyzed venous samples collected within 30 min of reperfusion from 127 KT recipients of both deceased donor (DD) and living donor (LD) allografts, with a longitudinal follow-up over 4 years. Nuclear and mitochondrial cfDNA were quantified and evaluated in relation to sC5b-9, NET markers, and allograft function. To further investigate the link between cell injury and complement activation, we incorporated an *in vitro* tubular cell necrosis model.

## 2 Materials and methods

### 2.1 Study population

This prospective cohort study included 127 KT recipients (41 LD and 86 DD) enrolled at Skåne University Hospital, Malmö, Sweden, from August 2018 to August 2020. All LD and 54 DD kidneys were preserved by static cold storage (SCS), and 32 DD kidneys underwent non-oxygenated hypothermic machine perfusion (HMP) (LifePort, Itasca, Illinois, United States). Eighty-two DD kidneys were from donation-after-brain-death (DBD) and four from donation-after-circulatory-death (DCD) donors.

Standard induction therapy included methylprednisolone and basiliximab, with maintenance immunosuppression of prednisolone, mycophenolate mofetil, and tacrolimus. One LD and 12 DD kidney recipients received thymoglobulin instead of basiliximab. Three LD kidney recipients received methylprednisolone-induction only. Eight recipients (4 LD and 4 DD) received rituximab with standard induction.

Donor/recipient characteristics and outcome data (plasma creatinine, DGF, graft failure, patient death, *de novo* donor-specific antibodies, and biopsy-proven acute rejections from indication biopsies) were obtained from patient records, the local transplant registry, and the ScandiaTransplant database. Follow-up was 48 months, with creatinine measured at 1, 3, 6, 12, 24, 36, and 48 months. Estimated glomerular filtration rate (eGFR, mL/min/1.73 m^2^) was calculated using the Lund-Malmö revised formula (10), and DGF was defined as dialysis within the first week. Written informed consent was obtained. The study was approved by the Regional and National Ethical Committee (DNR 2017/798, 2018/712). Baseline characteristics are presented in **Table 1**.

**Table 1:** Baseline characteristics.

Baseline characteristics				
Median (1^st^–3^rd^ quartile) or *n* (percent)		LD-KT (*n* = 41)	DD-KT (*n* = 86)	*P*-value
Sex—male		31 (75.6)	58 (67.4)	0.347
Recipient	Age	44.0 (35.0–54.0)	53.0 (45.0–61.0)	**0.002**
	BMI	26.3 (22.9–29.1)	25.4 (23.3–27.7)	0.728
Donor	Age	52.0 (47.0–56.0)	60.0 (47.0–68.0)	**0.015**
	BMI	26.5 (23.4–28.4)	25.7 (23.1–29.3)	0.898
Deceased donor type	DBD		82 (95.3)	N/A
	DCD		4 (4.7)	N/A
Cold ischemic time (minutes)		111.0 (78.0–127.0)	668.0 (535.0–792.0)	**<0.001**
Preformed donor-specific antibodies		6 (14.6)	6 (7.0)	0.200
Preservation method	SCS	41 (100.0)	54 (62.8)	**<0.001**
	Non-oxygenated HMP	0 (0.0)	32 (37.2)	**<0.001**
Kidney donor profile index			59.5 (34.5–78.0)	N/A
Induction therapy (methylprednisolone + additional)	Basiliximab	37 (90.2)	74 (86.0)	0.505
	Thymoglobulin	1 (2.4)	12 (14.0)	0.060
	Basiliximab + rituximab	4 (9.8)	4 (4.7)	0.271
	Methylprednisolone only	3 (7.3)	0 (0.0)	**0.032**
Pretransplant dialysis	Preemptive	17 (41.1)	11 (12.8)	**<0.001**
	Hemodialysis	15 (36.6)	47 (54.7)	0.057
	Peritoneal dialysis	9 (22.0)	28 (32.6)	0.219
Cause of kidney failure	Glomerulonephritis	23 (56.1)	25 (29.1)	**0.003**
	Polycystic kidney disease	7 (17.1)	13 (15.1)	0.777
	Diabetic nephropathy	2 (4.9)	12 (14.0)	0.224
	Hypertensive nephrosclerosis	1 (2.4)	12 (14.0)	0.060
	Alport syndrome	1 (2.4)	3 (3.5)	1.000
	Unknown	1 (2.4)	6 (7.0)	0.427
	Other	6 (14.6)	15 (17.4)	0.690

Continuous variables are expressed as median (1st to 3rd quartile) and categorical variables as *n* (percent). *P*-values for differences between LD and DD kidney transplant cases are presented. Bold text indicates *P*-values less than 0.05.

LD-KT, living donor kidney transplantation; DD-KT, deceased donor kidney transplantation; BMI, body mass index; DBD, donation after brain death; DCD, donation after circulatory death; non-oxygenated HMP, hypothermic machine perfusion; SCS, static cold storage.

### 2.2 Sample collection

Whole-blood samples (6 mL) were drawn using a 21G BD Vacutainer UltraTouch butterfly needle (BD Biosciences, Franklin Lakes, New Jersey, United States) into EDTA tubes (K2E BD Vacutainer, BD Biosciences, Franklin Lakes, New Jersey, United States). A baseline sample was drawn from the recipient’s external iliac vein pre-implantation, followed by transplant vein sampling at 1, 10, and 30 min post-reperfusion. Samples were immediately cooled in an ice sludge and centrifuged at 1,900×*g* for 10 min at 4°C, and the plasma was extracted and stored at −80°C. Sample aliquots were anonymized with numeric codes for blinded analysis.

### 2.3 Measurement of mitochondrial and nuclear cfDNA

Mitochondrial and nuclear cfDNA were quantified in samples from 127 KT recipients using droplet digital polymerase chain reaction (ddPCR) and expressed as copies/mL of plasma, normalized to plasma input volume. DNA was extracted using QIAamp DNA blood minikits (Qiagen, Hilden, Germany). Samples were fractioned into ~20,000 droplets by water–oil emulsion using a Bio-Rad QX200 droplet generator by adding sample, primers for mitochondrial (#10031253; Bio-Rad, Hercules, California, United States) and nuclear DNA (#10031244; Bio-Rad, Hercules, California, United States), restriction enzyme *Hin*dIII (# R0104S; New England Biolabs, Ipswich, Massachusetts, United States) mixed with NE buffer r2.1 (#B6002S; New England Biolabs, Ipswich, Massachusetts, United States) and Supermix for probes (#L002699C; Bio-Rad, Hercules, California, United States), and droplet oil (# 1863005; Bio-Rad, Hercules, California, United States) into separate DG8TM cartridge rows (# 1864008; Bio-Rad, Hercules, California, United States). Droplets were transferred to 96-well plates (# 12001925; Bio-Rad, Hercules, California, United States) and sealed with PCR thermal foil (#1814040; Bio-Rad, Hercules, California, United States) using a Bio-Rad px1 plate sealer, with the following thermocycling steps: activation at 95°C for 10 min, denaturation at 94°C for 30 s, 40 times 1-min cycles of annealing/extension at 60°C, held at 98°C for 10 min and then cooled to 4°C. Plates were stored overnight at 4°C before readout using droplet reader oil (#1863004; Bio-Rad, Hercules, California, United States) and a Bio-Rad QX200 droplet reader. Reference ranges for mitochondrial and nuclear DNA have not been established.

### 2.4 Assessment of neutrophil extracellular trap markers

NET markers were analyzed in 56 recipients (20 LD-KT and 36 DD-KT). Citrullinated histone H3 (#01620; Cayman Chemical, Ann Arbor, Michigan, United States) and human neutrophil elastase (HNE)-DNA (in-house method) were measured in 1:5 diluted plasma using enzyme-linked immunosorbent assays (ELISAs). Absorbance was read at 450/620 nm using a Cytation-5 multi-mode reader (BioTek, Winooski, Vermont, United States). Citrullinated histone H3 was analyzed per manufacturer’s protocol. For the HNE-DNA ELISA, NETs used as standard were generated by separating cells using Histopaque-11191 (#11191; Sigma-Aldrich, Saint Louis, Missouri, United States) and isolating neutrophils by Percoll (#17-0891-01; GE Healthcare, Chicago, Illinois, United States) gradient in peripheral venous blood from healthy donors (11). Neutrophils were incubated with phorbol myristate acetate (#P8139-5MG; Sigma-Aldrich, Saint Louis, Missouri, United States) for 4 h at 37°C and 5% CO_2_ to induce NETosis. NETs were detached with 1 U/mL of MNase (#EN0181; Thermo Scientific, Waltham, Massachusetts, United States) for 15 min at 37°C. For the ELISA, Nunc 96-well Maxisorp plates were coated overnight at 4°C with anti-neutrophil elastase rabbit polyclonal antibody (#481001; Merck Millipore, Burlington, Massachusetts, United States) diluted in PBS and blocked with blocking buffer (PBS with 1% BSA). NETs and patient plasma samples (diluted in blocking buffer) were incubated overnight at 4°C. Anti-DNA-POD antibody (component 2 of the Cell Death Detection kit; Roche, Basel, Swizerland) was added and incubated for 1 h at room temperature. The plate was developed with TMB and stopped with 0.5 M of H_2_SO_4_.

### 2.5 Assessment of soluble C5b-9

Soluble terminal complement activation fragment, sC5b-9, was assessed in the samples available from 58 recipients (23 LD-KT, 35 DD-KT) by an in-house ELISA as previously described (3).

### 2.6 Analyses of C5b-9 deposition on necrotic and live HK-2 cells by flow cytometry and confocal microscopy

Human proximal tubular cells (HK-2 cell line, #CRL-2190; ATCC, Manassas, Virginia, United States) were grown in keratinocyte serum-free medium, supplemented with bovine pituitary extract and human recombinant epidermal growth factor (#17005-042; Gibco, Waltham, Massachusetts, United States), at 37°C and 5% CO_2_. After detachment, the cells were washed twice with binding buffer (10 mM of HEPES, pH 7.4, 150 mM of NaCl, 5 mM of KCl, 1 mM of MgCl_2_, and 1.8 mM of CaCl_2_). To induce necrosis, cells were incubated at 56°C for 30 min. Necrotic cells were incubated at 37°C with 10% normal human serum (NHS, pool of six healthy donors) as a source of complement and in the presence of 5% CO_2_ to quantify the formation of C5b-9, using live cells as controls. NHS was collected from healthy volunteers following informed consent and according to ethical permit 2023–05543 from the Swedish Ethical Review Authority.

To quantify the deposition of C5b-9 with flow cytometry, the cells were washed once more with binding buffer after necrosis induction, and then approximately 300,000 necrotic cells/well and 100,000 live cells/well were added to the plate for 30 min of incubation with NHS diluted in binding buffer. Primary antibody mouse anti-human C9 aE11 (#HM2167; Hycult, Uden, Netherlands) and secondary antibody goat anti-mouse Alexa Fluor 405 (#A-31553; Thermo Fisher), diluted in PBS supplemented with 1% bovine serum albumin and 30 mM of NaN_3_, were added. Annexin AV-APC (#550474; BD Biosciences, Franklin Lakes, New Jersey, United States) and Via-Probe (#555816; BD Biosciences) diluted in binding buffer were used to gate live, apoptotic, and necrotic cells. After each step, the plate was incubated for 30 min at 4°C before washing. Flow cytometry was performed using CytoFLEX (Beckman Coulter, Brea, California, United States), and results were analyzed with FlowJo (Becton Dickinson & Company, Franklin Lakes, New Jersey, United States). As negative controls, binding of secondary antibody alone and HK-2 cells incubated without NHS were used.

To visualize C5b-9 deposition on HK-2 cells, confocal microscopy was used. The cells were incubated with NHS for 1 h and then washed with binding buffer. Live and necrotic cells were spun down with the Cellspin I cytocentrifuge (Tharmac, Limburg an der Lahn, Germany) on SuperFrost Plus Adhesion microscope slides (Epredia, Portsmouth, New Hampshire, United States) and then fixed with 4% formaldehyde. Samples were permeabilized and blocked with PBS supplemented with 1% bovine serum albumin, 30 mM of NaN_3_, and 0.1% Triton X-100 for 10 min at 37°C. Detection was performed using a mouse anti-human C9 aE11 antibody (#HM2167; Hycult, Uden, Netherlands) incubated for 1 h at 37°C and a donkey anti-mouse Alexa Fluor 488 secondary antibody (#715-546-151; Jackson Immunotools, West Grove, Pennsylvania, United States) incubated for 1 h at room temperature. To dilute antibodies and as wash in between steps, permeabilization/blocking buffer was used. Cells were mounted with mounting medium containing DAPI (#P36962; Invitrogen, Waltham, Massachusetts, United States). Images were captured with Zeiss LSM 800. As a negative control, HK-2 cells incubated without NHS were used.

### 2.7 Statistical analysis

Categorical variables are reported as frequencies (percentages), and continuous variables as medians (first–third quartile) or means (standard deviation), as appropriate. Nuclear and mitochondrial cfDNA levels are given in copies/mL, HNE-DNA in absorbance (nm), citrullinated histone H3 in ng/mL, and sC5b-9 in arbitrary units (AU)/mL. All markers were baseline-subtracted to account for pre-existing levels.

Pairwise comparisons used the Mann–Whitney *U*, chi-square, or Fisher’s exact tests, and one-way ANOVA was used for multiple comparisons. For outcome analyses, markers were summarized as individual area under the curve (AUC) values from 1-, 10-, and 30-min levels using the trapezoid method, capturing areas above pre-implantation levels. Associations with DGF used logistic regression and receiver operating characteristics (ROC) analysis. Longitudinal eGFR was summarized by time-weighted AUC or modeled using a linear mixed-effects model with a random intercept and autoregressive AR (1) covariance structure. K-means clustering and ROC with Youden’s *J* statistic defined thresholds of cold ischemic time (CIT) and 1-min cfDNA or sC5b-9. Spearman correlations assessed relationships between cfDNA, NET markers, and sC5b-9. Significance was set at *P <*0.05.

Results are presented as tables, dot plots, scatter plots, histograms, bar charts, and images. Analyses used IBM SPSS Statistics 28 (IBM Corp., Armonk, New York, United States), GraphPad Prism 10.4.1 (GraphPad Software), and FlowJo (Becton Dickinson & Company, San Diego, California).

## 3 Results

### 3.1 Baseline characteristics and general outcomes

Recipient and donor ages and CITs were lower in LD-KT than in DD-KT. Preemptive transplantations and glomerulonephritis were more frequent in LD-KT. Three LD-KT recipients received methylprednisolone-induction only. Apart from preservation methods, the remaining baseline characteristics were similar (**Table 1**).

Among DD-KT, CIT and the Kidney Donor Profile Index (KDPI) were comparable between SCS and non-oxygenated HMP kidneys [CIT: 668.0 min (544.0–767.0) versus 662.0 min (498.0–869.0), *P* = 0.939; KDPI 52.0% (20.0–78.0) versus 64.0% (55.0–78.0), *P* = 0.101, respectively].

In DD-KT, 12 patients developed DGF. Four patients died with functioning grafts: one on day 64 from tissue-invasive cytomegalovirus infection and three on days 939, 1,089, and 1,144 from undocumented causes. One DD-KT patient died with concurrent graft failure on day 774 from COVID-19. One case of early allograft artery thrombosis required transplantectomy on day 1 (not included as graft failure).

Three graft failures occurred in DD-KT due to acute antibody-mediated rejection, recurrent thrombotic microangiopathy, and an unknown cause on days 151, 396, and 1,397, respectively. One graft failure occurred in LD-KT on day 469 due to recurrent focal segmental glomerulosclerosis. Three LD-KT and one DD-KT patient were lost to follow-up after emigration on days 63, 69, 232, and 1,100, respectively.

At 4 years, eGFR, allograft and patient survival, rejection rates, and *de novo* donor-specific antibody frequencies were comparable between LD-KT and DD-KT (**Table 2**).

**Table 2:** General outcome parameters by 4 years post-transplantation.

General outcomes			
Outcomes by 4-years of follow-up (median [1^st^–3^rd^ quartile] and *n* [percent])	LD-KT	DD-KT	*P*-value
Delayed graft function	0 (0.0)	12 (14.0)	**0.009**
Non-death-censored graft failure	1 (2.4)	8 (9.3)	0.27
eGFR (mL/min/1.73 m^2^)	52.5 (40.5–69.0)	51.0 (37.1–66.5)	0.69
Biopsy-proven acute rejection	7 (17.1)	18 (20.9)	0.81
Patient death	0 (0.0)	5 (5.8)	0.32
*De novo* donor-specific antibodies	3 (7.3)	8 (19.5)	1.00

*P*-values for differences between LD and DD are presented. Bold text indicates *P*-values less than 0.05.

LD-KT, living donor kidney transplantation; DD-KT, deceased donor kidney transplantation; eGFR, estimated glomerular filtration rate.

### 3.2 Post-reperfusion releases of cfDNA and NET markers between modalities

**Figure 1:**
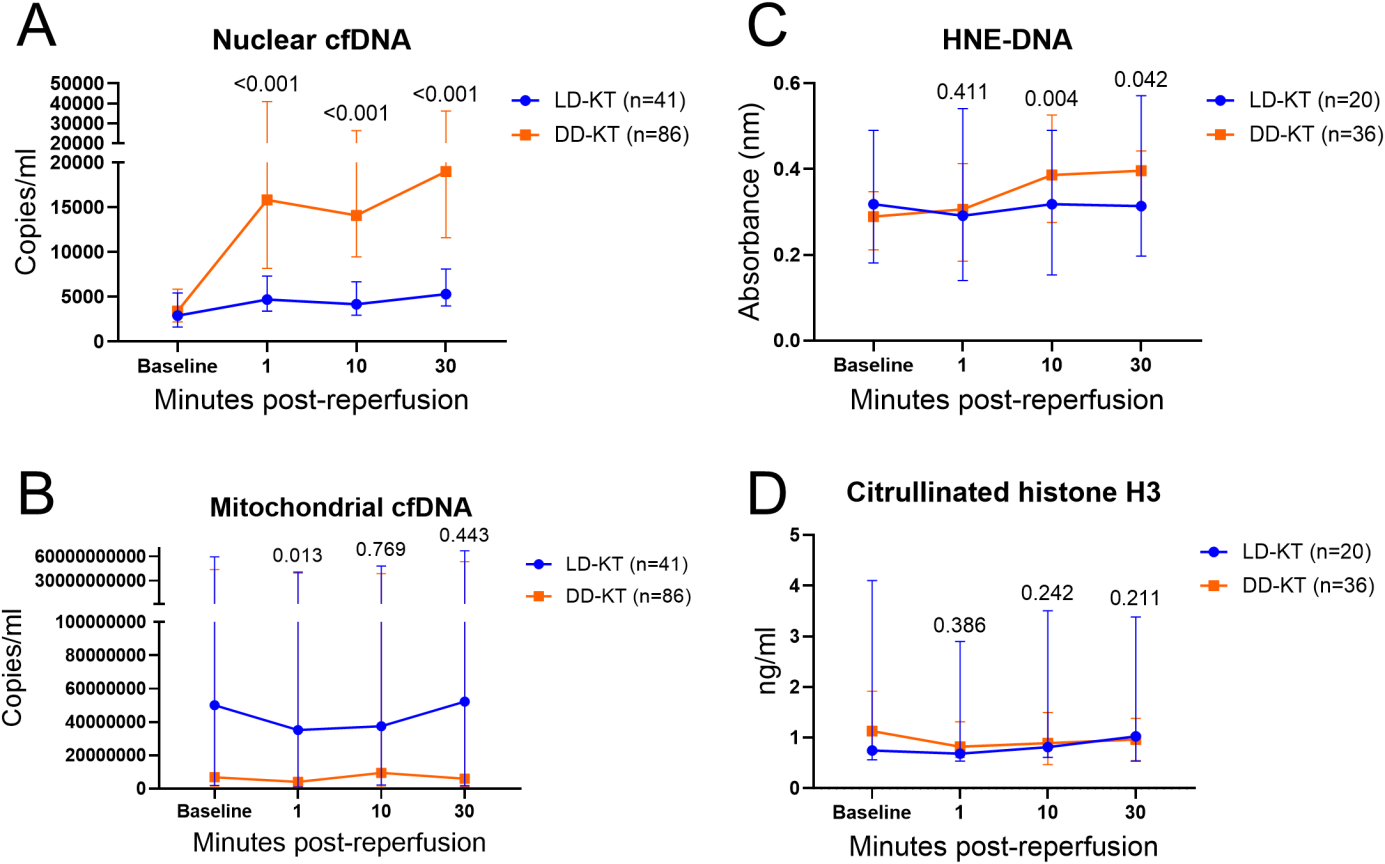
Pre- and post-reperfusion cfDNA and NET marker levels. Connected scatter plots depicting median (interquartile range) of cfDNA (LD-KT *n* = 41, DD-KT *n* = 86) and NET markers (LD-KT *n* = 20, DD-KT *n* = 36) by sampling time for LD (blue) and DD (orange) KT cases. To allow for visualization of baseline, plots present non-baseline-adjusted values for nuclear cfDNA **(A)**, mitochondrial cfDNA **(B)**, HNE-DNA **(C)**, and citrullinated histone H3 **(D)**. *P*-values are presented for differences in baseline-subtracted levels between modalities. LD-KT, living donor kidney transplantation; DD-KT, deceased donor kidney transplantation; cfDNA, cell-free DNA; HNE-DNA, human neutrophil elastase-DNA.

After reperfusion, DD kidneys exhibited higher levels of nuclear cfDNA compared to LD kidneys at 1, 10, and 30 min (*P* < 0.001 for all) (**Figure 1A**). Mitochondrial cfDNA levels were higher in DD kidneys at 1 min (*P* = 0.013), while no differences between modalities were observed for the 10- and 30-min levels (*P* = 0.769 and *P* = 0.443) (**Figure 1B**).

In the 56-patient subset analyzed for NET markers, HNE-DNA was higher in DD-KT at 10 and 30 min (*P* = 0.004 and *P* = 0.042, respectively), but not at 1 min (*P* = 0.411) (**Figure 1C**). Citrullinated histone H3 did not differ between modalities at any time point (*P* = 0.386, *P* = 0.242, and *P* = 0.211) (**Figure 1D**).

HNE-DNA correlated with nuclear cfDNA levels at 10 and 30 min (*ρ* 0.607, *P* < 0.001 and *ρ* 0.475, *P* < 0.001, respectively) and with mitochondrial cfDNA levels at 1 min (*ρ* 0.340, *P* = 0.011). Citrullinated histone H3 displayed a correlation with mitochondrial cfDNA at 30 min (*ρ* 0.285, *P* = 0.037), but none were observed with nuclear cfDNA. No correlations were observed between HNE-DNA and citrullinated histone H3 throughout the reperfusion phase. Correlation tables are provided in **Supplementary Tables S1**, **S2**.

### 3.3 Association of cfDNA release and DGF

To evaluate the association of cfDNA types with DGF in the entire study population, cumulative cfDNA levels (AUCs) were log10-transformed to reduce scale skewness for regression analysis, while untransformed AUCs were used for pairwise and ROC analyses. The day-1 transplantectomy case was excluded.

**Figure 2:**
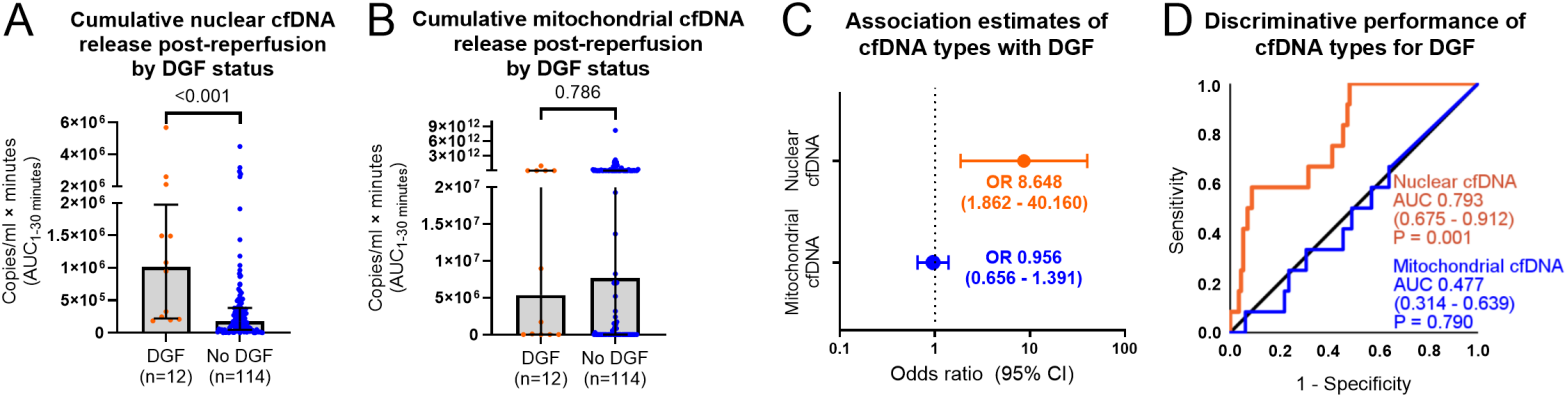
Nuclear and mitochondrial cfDNA and associations with delayed graft function. Plots presenting differences in cumulative cfDNA release post-reperfusion (AUC_1–30 minutes_) stratified by DGF status (DGF *n* = 114, no DGF *n* = 12) by Mann–Whitney *U* tests **(A, B)**, association estimates of cumulative cfDNA release with DGF by binary logistic regression **(C)**, and discriminative performance of cumulative cfDNA release for DGF by receiver operating characteristic curves **(D)**. Cumulative post-reperfusion release of nuclear and mitochondrial cfDNA is represented by individual AUCs from 1-, 10-, and 30-min post-reperfusion levels after baseline subtraction. For binary logistics regression, the AUCs of cfDNA types were log10-transformed. Bar charts present median with scatter distribution and error bars of interquartile range; the dot plot presents OR with 95% confidence interval. *P*-values are presented for the specified analyses. cfDNA, cell-free DNA; OR, odds ratio; DGF, delayed graft function; AUC, area under the curve.

Higher nuclear cfDNA levels were observed in DGF cases (*n* = 12) versus non-DGF cases (*n* = 114) [1,015,375 copies/mL × min (228,625–1,812,112) versus 175,950 copies/mL × min (47,763–380,472), respectively, *P* < 0.001] (**Figure 2A**). Mitochondrial cfDNA levels showed no difference by DGF status (*P* = 0.786) (**Figure 2B**).

Nuclear cfDNA levels were significantly associated with DGF [8.648 OR (95% CI 1.862–40.160), *P* = 0.006], indicating that each 10-fold increase in cumulative nuclear cfDNA release between 1 and 30 min conferred an 8.6-fold increase in odds of DGF. No significant association was found between mitochondrial cfDNA and DGF (*P* = 0.812) (**Figure 2C**).

ROC analysis indicated that nuclear cfDNA release distinguished DGF from non-DGF cases (ROC AUC 0.793, 95% CI 0.675–0.912, *P* = 0.001), while mitochondrial cfDNA release did not (*P* = 0.790) (**Figure 2D**).

### 3.4 Association of cfDNA release and allograft function over the long-term follow-up

A linear mixed-effects model was used to assess associations between cfDNA levels and allograft function over 4 years. To meet normality assumptions, individual AUCs of cfDNA levels were log10-transformed. Exclusions from this assessment included graft failures, patient deaths, patients lost to follow-up within the first year, and the day-1 transplantectomy case. Remaining graft failures were assigned an eGFR of 10 mL/min/1.73 m² for the remainder of the follow-up, while the last eGFR values were carried forward for patients lost to later follow-up or who died with a functioning graft after 1 year.

In this model, neither nuclear nor mitochondrial cfDNA release showed significant associations with eGFR (*P* = 0.722 and *P* = 0.276) (**Table 3**).

**Table 3:** Longitudinal allograft function and cfDNA release.

Long-term impact of cfDNA on allograft function (eGFR) over 4 years of follow-up			
Parameter	Estimate	*P*-value	95% CI
Nuclear cfDNA (log10 AUC)	1.074	0.722	−4.925 to 7.074
Mitochondrial cfDNA (log10 AUC)	−1.071	0.276	−3.016 to 0.874

Linear mixed-effects model on the long-term association between baseline-subtracted AUC cfDNA types (log10-transformed) and eGFR levels over a 4-year follow-up (repeated eGFR values from 1, 3, 6, 12, 24, 36, and 48 months post-transplantation). A random intercept per patient accounts for individual variability.

cfDNA, cell-free DNA; eGFR, estimated glomerular filtration rate; AUC, area under the curve.

### 3.5 Assessments on sC5b-9 associations with cfDNA and allograft function over the long-term follow-up

In our previous work, persistent generation of the thromboinflammatory marker sC5b-9, defined as detectable levels at 30 min post-reperfusion, was associated with 2-year allograft dysfunction (3). Of the 63 recipients in that study, 58 were included in the current cohort. To assess the relationship between post-reperfusion cfDNA release and thromboinflammation, we analyzed the corresponding cfDNA and sC5b-9 levels in this subset, comparing recipients with persistent sC5b-9 generation (*n* = 8) and those without (*n* = 49). One patient (LD-KT) lacking 30-min sC5b-9 data was excluded.

Spearman correlations showed that nuclear cfDNA significantly correlated with sC5b-9 levels from 1 min (*ρ* 0.460, *P* < 0.001) to 10 min (*ρ* 0.373, *P* = 0.004), while mitochondrial cfDNA correlated at 10 min (*ρ* 0.286, *P* = 0.031) (**Supplementary Table S3**).

**Figure 3:**
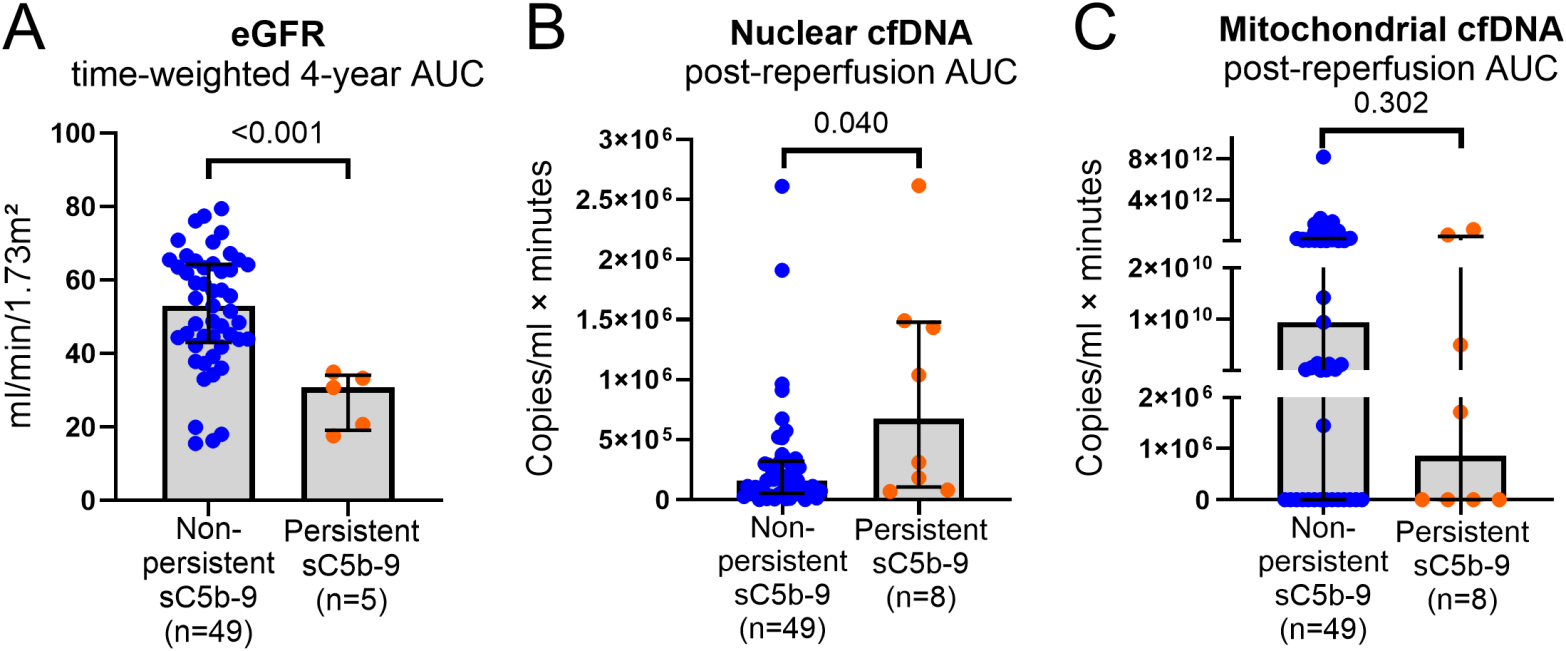
Soluble C5b-9 generation in relation to allograft function over 4 years and cfDNA release. Bar charts with scatter presenting distributions of time-weighted average of 4-year eGFR (AUC of 1, 3, 6, 12, 24, 36, and 48 months post-transplantation, divided by 48 months) **(A)**, post-reperfusion AUCs of nuclear **(B)** and mitochondrial **(C)** cfDNA (from baseline-subtracted 1-, 10- and 30-min levels) stratified by persistent sC5b-9 status (denoted in blue and orange). Persistent sC5b-9 was defined as detectable sC5b-9 generation at 30 min post-reperfusion. Sample sizes were *n* = 49 and *n* = 5 for non-persistent and persistent sC5b-9 in **(A)** and *n* = 49 and *n* = 8, respectively, in **(B, C)**. Bars depict the median, and error bars the interquartile range. *P*-values are presented for differences by Mann–Whitney *U* tests. eGFR, estimated glomerular filtration rate; cfDNA, cell-free DNA; sC5b-9, soluble C5b-9; AUC, area under the curve.

The 4-year time-weighted average eGFR, calculated as the AUC of longitudinal eGFR values throughout 1–48 months divided by 48, was lower in recipients with persistent sC5b-9 generation (*n* = 5) compared to those without (*n* = 49) [30.78 mL/min/1.73 m^2^ (IQR 20.68–33.31) versus 53.06 mL/min/1.73 m^2^ (IQR 43.88–64.19), *P* < 0.001] (**Figure 3A**).

Cumulative nuclear cfDNA release (AUC from 1 to 30 min) was higher in recipients exhibiting persistent sC5b-9 generation (*n* = 8) compared to those without (*n* = 49) [674 861.5 copies/mL × min (IQR 130,850–1,463,199) versus 161,112 copies/mL × min (IQR 61,009–318,500), *P* = 0.040] (**Figure 3B**), whereas cumulative mitochondrial cfDNA levels did not differ by 30-min sC5b-9 status (*P* = 0.302) (**Figure 3C**).

### 3.6 Comparison of nuclear cfDNA and sC5b-9 in DD kidneys by preservation methods

To explore whether the organ preservation method influenced early intragraft injury and inflammatory activation, we compared nuclear cfDNA and sC5b-9 kinetics between DD kidneys preserved by non-oxygenated HMP and SCS.

Nuclear cfDNA levels (copies/mL) did not differ between recipients of non-oxygenated HMP kidneys (*n* = 32) and SCS kidneys (*n* = 54) at 1 min [8,300.00 (IQR 2,478.80–26,685.03) versus 13,996.76 (IQR 4,258.32–47,919.32), *P* = 0.106], 10 min [8,150.00 (IQR 4,198.02–16,010.01) versus 10,748.57 (IQR 4,400.00–23,093.54), *P* = 0.348], and 30 min [12,446.56 (IQR 5,842.14–24,329.50) versus 14,000.00 (IQR 6,749.56–26,321.67), *P* = 0.409] (**Supplementary Figure S1**). Additionally, the cumulative nuclear cfDNA release [copies/mL × min (AUC_1–30 min_)] was comparable between DD-KT cases by non-oxygenated HMP and SCS preservation [279,025.00 (IQR 164,924.00–581,680.00) versus 358,439.00 (IQR 198,748.00–946,954.00), *P* = 0.221].

In recipients of DD kidneys with available sC5b-9 measurements (non-oxygenated HMP *n* = 15, SCS *n* = 20), baseline-subtracted sC5b-9 levels (AU/mL) were lower in recipients of non-oxygenated HMP kidneys compared to SCS kidneys at 1 min [0.00 (IQR 0.00–3.02) versus 4.39 (IQR 0.37–11.03), *P* = 0.033]. Generation of sC5b-9 at 10 and 30 min was only detected in 0 and 1 recipients of non-oxygenated HMP kidneys, respectively, compared to 9 and 5 in the SCS group, precluding meaningful non-parametric testing due to minimal non-oxygenated HMP group size. However, the cumulative sC5b-9 release [AU/mL × min (AUC_1–30 min_)] was significantly lower in recipients of non-oxygenated HMP kidneys compared to SCS kidneys [0.00 (IQR 0.00–13.59) versus 54.26 (IQR 12.83–112.20), *P* < 0.001].

### 3.7 Immediate release of cfDNA and sC5b-9 by CIT

To assess the impact of ischemia on immediate cfDNA and sC5b-9 release, we evaluated CIT in relation to 1-min post-reperfusion levels.

**Figure 4:**
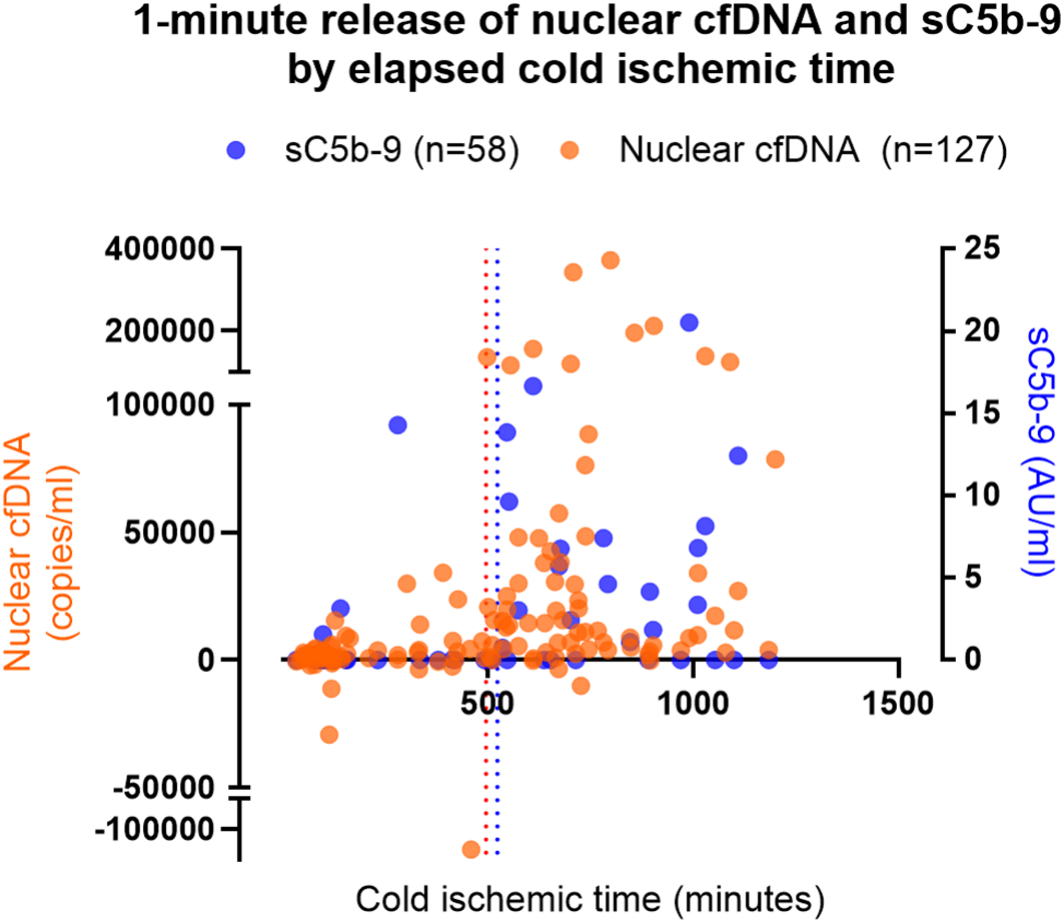
One-minute release of nuclear cfDNA (*n* = 127) and sC5b-9 (*n* = 58) by cold ischemic time. Scatter plot presenting baseline-subtracted 1-min release of nuclear cfDNA (orange) and sC5b-9 (blue) by elapsed cold ischemic time. Orange and blue vertical dotted lines indicate Youden’s *J* estimated cutoffs from receiver operating characteristics for increased 1-min release at 495.0 and 522.5 min of cold ischemic time, respectively. cfDNA, cell-free DNA; sC5b-9, soluble C5b-9.

K-means clustering (*k* = 2) identified two distinct clusters of 1-min nuclear cfDNA at 190,678 and 9,700 copies/mL (*P* < 0.001) with the corresponding CIT of 774.8 and 477.3 min (*P* = 0.004), respectively. ROC analysis on the higher cluster state and CIT (ROC AUC 0.772, 95% CI 0.657–0.886, *P* = 0.004), followed by Youden’s *J* statistic, revealed an estimated 495.0-min CIT cutoff for increased nuclear cfDNA release (100% sensitivity, 42% specificity) (**Figure 4**). No significant paired clustering of 1-min mitochondrial cfDNA and corresponding CIT was found (*P* < 0.001 and *P* = 0.557, respectively), and no further cutoff estimation was attempted.

Among 58 recipients with sC5b-9 data, ROC analysis of 1-min sC5b-9 expression by CIT (ROC AUC 0.771, 95% CI 0.647–0.895, *P* = 0.001) and the subsequent Youden’s *J* statistic identified a CIT cutoff at 522.5 min (82% sensitivity, 72% specificity) for immediate sC5b-9 release (**Figure 4**).

### 3.8 Cell culture necrosis model and C5b-9

A necrosis model on human proximal tubular cells (HK-2) was incorporated to further elaborate on possible mechanistic links from findings of increased nuclear cfDNA release, predominantly in DD kidneys, and correlations with elapsed CIT and sC5b-9.

**Figure 5:**
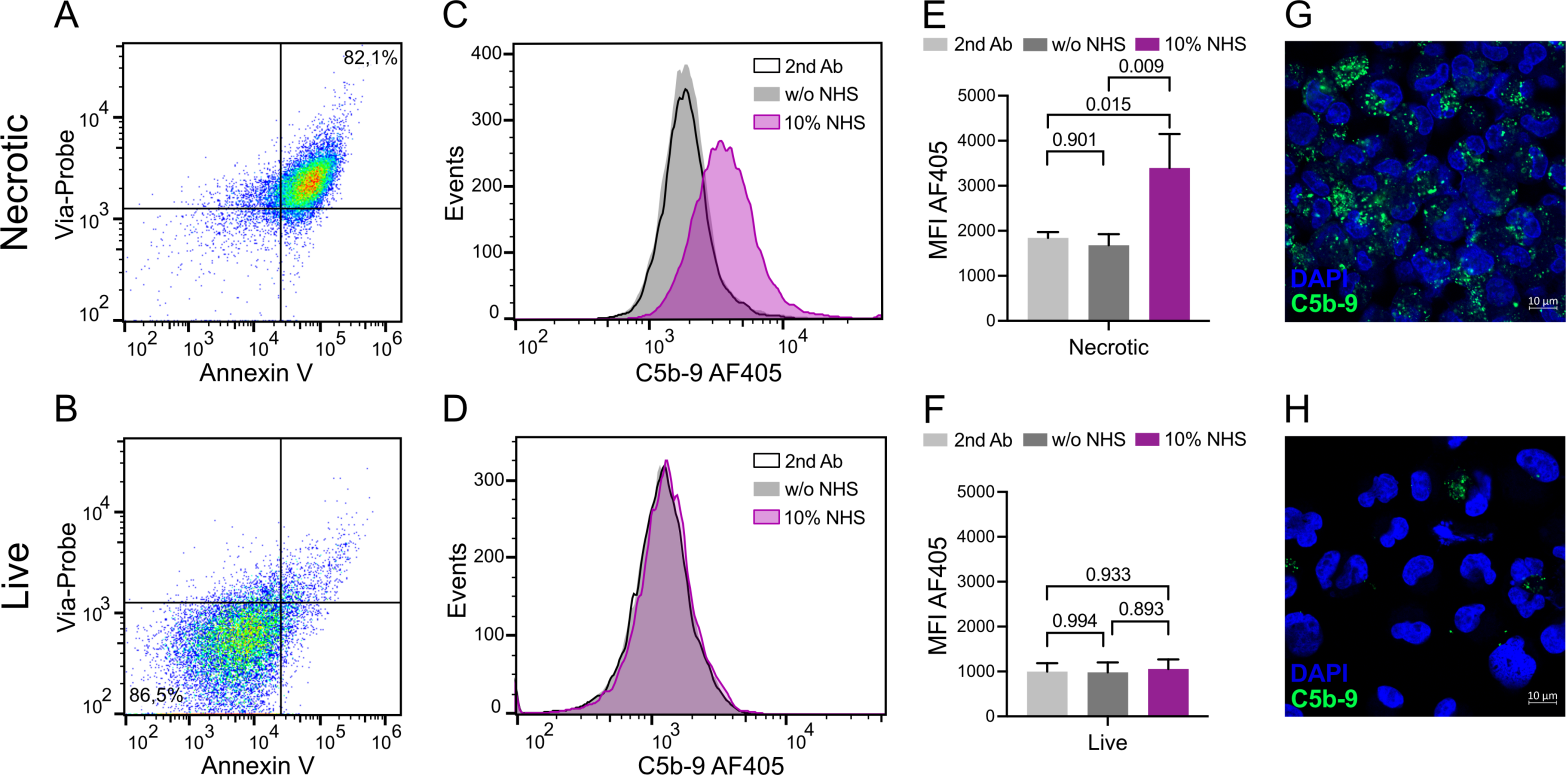
C5b-9 deposition on live and necrotic HK-2 cells assessed by flow cytometry and confocal microscopy. Annexin V and Via-Probe were used to gate live (AV-, Via-) and necrotic cells (AV+, Via+) **(A, B)**. C5b-9 deposition was determined on cells incubated with buffer only or NHS. Cells incubated with NHS but without primary antibody were used as an additional negative control. Representative histograms are presented **(C, D)**. MFI of *n* = 3 repeats was used for comparison between the necrotic cells and negative controls **(E)** and live cells and negative controls **(F)**. Graphs depict mean with standard deviation. *P*-values from one-way ANOVA are presented **(E, F)**. C5b-9 deposition (green) on live and necrotic HK-2 cells visualized by confocal microscopy. Nuclei were counterstained with DAPI (blue). Scale bar corresponds to 10 µm **(G, H)**. w/o, without; NHS, normal human serum; MFI, mean fluorescence intensity.

After inducing necrosis, cells were assessed for deposited C5b-9 via flow cytometry and confocal microscopy. Heat-induced necrotic cells and live cells were gated according to Annexin-V and Via-Probe characteristics (**Figures 5A, B**) and incubated with buffer alone or supplemented with 10% NHS. A C9 neo-antigen antibody was used to detect C5b-9 deposition by flow cytometry (**Figures 5C, D**). Necrotic cells incubated in NHS showed significantly more deposition of C5b-9 complexes than cells incubated with buffer alone, whereas no difference and low depositions were detected on live cells (**Figures 5E, F**). Cells incubated with NHS but without primary antibody served as an additional negative control.

C5b-9 deposition on live and necrotic cells was also visualized by confocal microscopy (**Figures 5G, H**). Staining for C5b-9 (green) was markedly more extensive on necrotic cells (**Figure 5G**) compared to live controls (**Figure 5H**).

### 3.9 Censoring for thymoglobulin induction

Exclusion of KT cases with thymoglobulin induction (*n* = 13) did not affect the results of the presented cfDNA analyses. These cases were therefore included to retain statistical power.

### 3.10 Literature search

To contextualize our findings, we conducted a targeted literature review assessing cfDNA in the immediate and early post-transplant period. PubMed/MEDLINE and Embase were searched using free-text terms and MeSH terms for the keywords kidney, renal, (kidney) transplantation, cell-free, circulating, free, and DNA. Searches yielded 461 and 828 results from PubMed/MEDLINE and Embase, respectively. Studies were included if they assessed cfDNA within 3 weeks post-transplantation and reported associations with allograft function, excluding those focused solely on rejection. Eleven relevant studies were identified and summarized in **Table 4**.

**Table 4:** Summary of KT studies assessing early postoperative cell-free DNA types in relation to allograft function.

Author and year	Investigation (in short)	Study size	cfDNA type	Sampling compartment and timing	Findings concerning early sampling and allograft function
Present study	Intraoperative cfDNA dynamics during reperfusion	*n* = 127	Mitochondrial and nuclear cfDNA	Plasma (allograft venous effluent) at: pre-implantation and 1, 10, and 30 min post-reperfusion	Cumulative 1- to 30-min post-reperfusion nuclear cfDNA release associated with DGF, but not with long-term (4-year) allograft function.
Cucchiari et al., 2023 (12)	Early dd-cfDNA evolution after transplantation	*n* = 61	dd-cfDNA	Plasma (systemic) at: 24 h post-transplantation and POD 7, 14, 30	24-h dd-cfDNA fractions associated with functional DGF and lower 6-month eGFR. 7-day dd-cfDNA correlated with dialysis duration in DGF cases and reduced 7-year iBox allograft survival probability.
Kim et al., 2019 (13)	Mitochondrial cfDNA in the early post-transplant period	*n* = 85	Mitochondrial and nuclear cfDNA	Plasma (systemic) and urine at: POD ~17	Urinary mitochondrial cfDNA concentrations associated with DGF and renal recovery time. Lower urinary mitochondrial cfDNA linked to higher eGFR at 9 and 12 months.
Shen et al., 2019 (14)	dd-cfDNA in the early post-KT period between modalities and DGF status	*n* = 21	dd-cfDNA	Plasma (systemic) at: 3 h post-transplantation and POD 1–7, 10, 14	dd-cfDNA fractions at 3 h and POD 1 positively correlated with serum-creatinine. No differences in dd-cfDNA fractions were observed between the DGF and non-DGF groups between 3 h and POD 7.
Gielis et al., 2018 (15)	Kinetics of dd-cfDNA in stable KT cases in the early post-transplantation period	*n* = 107	dd-cfDNA	Plasma (systemic) at: POD 1 + 3 and weeks 1–4, 6, 8, 10, and 3 months	dd-cfDNA fractions from days 1 to 7 correlated with proteinuria and a sampling closer to the transplantation date. dd-cfDNA fraction kinetics within the first 10 days were not associated with DGF occurrence. No correlation found between dd-cfDNA fractions within POD 10 and allograft function.
Lane, Nie, Kayler. 2022 (16)	The clinical validity of dd-cfDNA as an allograft injury sensor	*n* = 71	dd-cfDNA	Plasma (systemic) at: POD 14–37	High dd-cfDNA fractions (≥0.5%) were not associated with DGF or 1-year eGFR ≤40 mL/min/1.73 m^2^.
Kusaka et al., 2023 (17)	Total cfDNA levels in relation to DGF	*n* = 30	Total cfDNA	Plasma (systemic) at: POD 3 + 5	Total cfDNA levels increased in DGF cases and correlated with DGF duration.
Jansen et al., 2020 (18)	Plasma and urine mitochondrial DNA as diagnostic markers of DGF	*n* = 43	Total cfDNA and mitochondrial cfDNA	Plasma (systemic) and urine at: POD <14 (mean ~8 days)	Urinary mitochondrial and non-coding cfDNA correlated with concurrent plasma creatinine levels. Higher urinary mitochondrial cfDNA concentrations associated with DGF. Selected genes in urinary mitochondrial cfDNA showed diagnostic performance for DGF.
Wei et al., 2024 (19)	dd-cfDNA as an indicator for evaluating early allograft status	*n* = 138	dd-cfDNA	Plasma (systemic) at: POD 1 + 7	dd-cfDNA concentration and fraction changes between POD 1 and 7, and 7-day dd-cfDNA concentrations, correlated inversely with 1–2-year eGFR.
Yang et al., 2022 (20)	Outlier dd-cfDNA levels in the initial post-transplantation phase	*n* = 230	dd-cfDNA	Plasma (systemic) at: POD 1	Higher POD 1 dd-cfDNA concentrations associated with elevated POD 7 serum-creatinine.
Di et al., 2021 (21)	dd-cfDNA as a predictor of renal allograft function	*n* = 87	dd-cfDNA	Plasma (systemic) at: POD 1, 7, 14-20, 30–45	POD 1 dd-cfDNA fractions were higher in DGF cases. Fractions <1% were associated with a lower probability of eGFR <60 at POD 90. Day 90 eGFR predictions by dd-cfDNA matched observed outcomes.
Nie et al., 2022 (22)	Early dynamics of ddcfDNA in pediatric KT	*n* = 21	dd-cfDNA	Plasma (systemic) at: POD 1, 4, 7, 14, 30, 60, 90	Early dd-cfDNA fraction dynamics were not associated with DGF.

dd-cfDNA, donor-derived cell-free DNA; POD, postoperative day; DGF, delayed graft function; eGFR, estimated glomerular filtration rate; KT, kidney transplantation.

## 4 Discussion

We investigated the immediate release of nuclear and mitochondrial cfDNA following kidney allograft reperfusion in 127 transplant recipients to assess its relationship with IRI and transplant outcomes. Cell-free DNA levels were quantified in DD and LD transplant cases, alongside sC5b-9 as a marker of thromboinflammation and NET markers. The principal finding was a prompt and sustained release of nuclear cfDNA, predominantly observed in DD kidneys, correlating with ischemic burden and early allograft dysfunction.

The clinical utility of cfDNA as a biomarker for detecting allograft injury has gained increasing attention in recent literature. Most KT studies to date have particularly focused on donor-derived cfDNA monitoring during post-transplant follow-up, where it has shown diagnostic value for rejection episodes (23–27) and, in some reports, predictive value for long-term allograft function and survival (12, 27). However, as outlined in **Table 4**, studies concerning early postoperative cfDNA in relation to early allograft injury or longer-term function have mainly relied on systemic sampling performed no earlier than 24 h post-transplantation, which precludes direct assessment of the immediate cfDNA dynamics initiated by reperfusion. To our knowledge, this is the first study to quantify both nuclear and mitochondrial cfDNA directly in allograft venous effluent during the first 30 min of reperfusion, providing direct insight into early intragraft injury.

In this setting, DD kidneys released a marked early surge of nuclear cfDNA compared to LD kidneys (**Figure 1A**), where the release magnitude (baseline-subtracted AUCs from 1 to 30 min) was associated with DGF (**Figure 2**). This early cfDNA release, predominant in DD-KT recipients, alongside loss of early graft function, likely reflects IRI-induced cell injury, which is known to activate necrotic cell death pathways (28, 29).

Supporting the link between ischemic burden and nuclear cfDNA release, we observed a clear relationship between cumulative CIT and nuclear cfDNA levels immediately post-reperfusion, with a distinct inflection point at 495 min (**Figure 4**). Previous findings from our group have shown that reperfusion of ischemic kidneys triggers prompt engagement of the intravascular innate immune network, encompassing the complement, coagulation, and kinin–kallikrein pathways and, by extension, a thrombo-inflammatory response. Among the measurable components of this response, sC5b-9 emerged as an integrative marker of thromboinflammation, given its strong correlation with upstream mediators across these pathways (3).

To further explore the relationship between cfDNA release and thromboinflammation, we analyzed sC5b-9 levels in a subset of 58 recipients. In this subgroup, we identified a corresponding inflection point in sC5b-9 generation at 522.5 min of CIT, closely aligning with the nuclear cfDNA threshold (**Figure 4**). The convergence of these thresholds, together with a consistent correlation between nuclear cfDNA and sC5b-9 at 1 and 10 min (**Supplementary Table S3**), supports a coordinated response linking acute cell injury and thromboinflammatory activation during early reperfusion.

In line with these observations, nuclear cfDNA was associated with early allograft dysfunction but not with long-term function (**Table 3**), consistent with its role as a marker of early IRI-related injury. This pattern aligns with recent clinical KT observations showing that early donor-derived cfDNA elevations at 24 h post-reperfusion were associated with DGF, whereas its persistence beyond the first week was more strongly linked to impaired long-term allograft outcomes (12). In our cohort, patients with persistent sC5b-9 generation at 30 min in the subset of those profiled for thromboinflammation exhibited higher cumulative nuclear cfDNA release and lower eGFR at 4 years (**Figures 3A, B**), suggesting that downstream inflammatory response may contribute to concomitant injury beyond the initial reperfusion phase, with possible implications for long-term outcome trajectory.

To explore whether this response is modifiable, we next compared the effects of the organ preservation method. In the DD-KT subgroup, organ preservation with non-oxygenated HMP was associated with reduced levels of sC5b-9 compared with SCS, while nuclear cfDNA release remained similar. These findings suggest that non-oxygenated HMP may not prevent the acute parenchymal injury that occurs at the moment of reperfusion, an injury whose severity is likely conditioned by preceding conditions such as prolonged CIT, but may instead attenuate downstream inflammation following reperfusion, through mechanisms distinct from direct structural cell protection.

Recently, we characterized early inflammatory processes during the first 30 min of reperfusion using proteomic profiling. In DD kidneys, this analysis revealed a post-reperfusion surge in hepatocyte growth factor (HGF), a molecule with high affinity for heparan sulfate proteoglycans within the endothelial glycocalyx. We argued that the prompt surge in circulating HGF following reperfusion likely reflects shedding or disruption of the glycocalyx barrier, consistent with acute structural and endothelial injury at reperfusion. Notably, this was followed by a progressive increase in interleukin-33 peaking at 30 min post-reperfusion and reflecting ongoing downstream inflammatory activation (30). This sequential pattern of acute cell injury followed by inflammatory signaling seems to parallel the cfDNA and sC5b-9 kinetics observed in the present study. Collectively, these findings suggest that while non-oxygenated HMP may not prevent the initial insult inflicted at reperfusion, it may mitigate subsequent downstream inflammatory cascades, underscoring the multiphasic nature of IRI in DD kidneys. To further support the observed link between tissue injury and inflammation, we used an *in vitro* model in which necrosis was induced in human proximal tubular epithelial cells (**Figure 5**). Necrotic cells are known to release cfDNA and facilitate the formation and deposition of C5b-9 (31). Consistent with this mechanism and our clinical observations, conformationally changed C9, as in C5b-9, was detected selectively on necrotic, but not viable, tubular cells. While this might not establish causality *in vivo*, it supports the plausibility that cell injury during renal allograft IRI may contribute to thromboinflammatory downstream activation.

Beyond cell injury, NET formation has been proposed as a potential cfDNA source in IRI (8, 9). However, our analysis did not reveal a consistent pattern supporting NETs as a major contributor in this setting. Although HNE-DNA levels were elevated at 10 and 30 min post-reperfusion in DD kidneys, citrullinated histone H3, a well-established NET marker (32, 33), did not differ between transplant modalities (**Figures 1C, D**). Moreover, no correlations were found between NET markers themselves (**Supplementary Table S2**). NETs are increasingly recognized in renal IRI (34), but their dynamics differ from the immediate cfDNA release observed here. Experimental models show that substantial NET accumulation arises hours after reperfusion, peaking approximately 6–24 h, rather than within the first minutes (35, 36). Moreover, NETosis in IRI is highly compartmentalized, occurring predominantly in the tubulointerstitial space and peritubular capillaries as neutrophils extravasate into injured tissue (37). In this context, our intraoperative sampling window, capturing the earliest intravascular events, likely reflects parenchymal necrosis as the dominant source of nuclear cfDNA, while tissue-localized NET activity may develop later and be incompletely represented in circulating markers at this earliest post-IRI window. In contrast to nuclear cfDNA, mitochondrial cfDNA showed no associations with allograft dysfunction and no clear temporal variation post-reperfusion between kidneys of different donor types, except for lower 1-min levels in LD-KT—likely reflecting higher baseline values (**Figure 1B**). In our setting, mitochondrial cfDNA appeared less robust as a marker of acute injury compared to nuclear cfDNA. A similar finding was reported in trauma patients, where ddPCR measurements of only nuclear, but not mitochondrial, cfDNA correlated with inflammation and adverse outcomes (38). This discrepancy may reflect biological differences in how these DNA types are released and processed. Nuclear cfDNA is typically released in fragments during cell death, making it a more specific direct indicator of tissue injury (39). In contrast, mitochondrial DNA can also be released in response to cellular stress or through active secretion mechanisms (40), which could help explain the elevated baseline levels observed in our study. These factors may contribute to the weaker association between mitochondrial cfDNA and inflammation or allograft function.

Notably, prior studies linking mitochondrial cfDNA to transplant outcomes analyzed systemic plasma or urine samples at time points well separated from the reperfusion event, likely reflecting downstream or cumulative injury (13, 18, 41, 42). In contrast, our renal vein sampling within 30 min of reperfusion offers a direct window into the immediate intragraft response to IRI.

This study has limitations. First, our cfDNA sequencing did not distinguish donor-derived from recipient-derived cfDNA. However, the experimental setting strongly suggests that the observed surge in nuclear cfDNA originates from the allograft, given the prompt and substantial increase at 1 min post-reperfusion in allograft venous return compared to systemic baseline levels. Second, the absence of concurrent biopsies limits our ability to directly correlate cfDNA levels with histopathological evidence of allograft injury. Third, the study was conducted at a single center with a cohort characterized by relatively short CITs in DD-KT (median 11.1 h), which may limit the generalizability of findings to settings with longer ischemic exposures. Fourth, although cfDNA surge paralleled early thromboinflammation, these data do not establish cfDNA as a causal driver of inflammatory activation; cfDNA is best interpreted here as a marker of parenchymal injury, and future mechanistic studies are needed to determine potential contributory roles. Fifth, while this study provides high-resolution insight into the earliest phase of reperfusion injury, it does not define cfDNA thresholds or ranges that could currently guide clinical decision-making. Moreover, although early nuclear cfDNA release was associated with DGF, it was not associated with long-term allograft function, indicating that cfDNA at this stage primarily reflects the severity of the immediate reperfusion insult rather than the later immunological and reparative processes that influence chronic allograft trajectory. Whether nuclear cfDNA release at reperfusion will have future clinical utility remains to be determined.

Despite these limitations, the study has several notable strengths. It draws on a well-characterized cohort of 127 kidney transplants with 4 years of follow-up and employs intraoperative renal venous sampling, enabling high-resolution assessment of cfDNA dynamics during the critical early reperfusion phase. In addition, the parallel assessment of thromboinflammation and NETs, along with a supportive *in vitro* model of epithelial cell injury, provides a biological context and reinforces the plausibility of the observed associations.

In summary, this study refines current understanding of IRI in clinical KT by providing high-resolution, real-time evidence of the earliest intragraft events at reperfusion. We show that DD kidneys experience an immediate surge in nuclear cfDNA following reperfusion, reflecting acute parenchymal injury occurring alongside early thromboinflammation. The associations between prompt cfDNA release, thromboinflammation, and DGF emphasize the critical importance of the initial minutes after reperfusion and support the importance of pre-emptive interventions targeting this phase. Furthermore, our comparative analysis further indicates that non-oxygenated HMP does not prevent the immediate tissue injury inflicted at reperfusion but may attenuate subsequent inflammatory activation. Future studies are needed to clarify whether early cfDNA dynamics can meaningfully contribute to individualized risk assessment, help inform organ preservation strategies, or serve as a readout for evaluating emerging interventions aimed at modulating IRI. Finally, the demonstrated safety and feasibility of serial renal-venous sampling establish it as a practical clinical tool for monitoring early allograft injury and assessing future IRI-modifying interventions.

## Data Availability

A dataset including raw data and selected clinical variables is registered within Region Skåne’s official records system. Access requests will be reviewed in accordance with the Swedish Public Access to Information and Secrecy Act (OSL). Requests to access the datasets should be directed to diariet@skane.se.

## References

[1] Zhao H Alam A Soo AP George AJT Ma D . Ischemia-reperfusion injury reduces long term renal graft survival: mechanism and beyond. EBioMedicine. (2018) 28:31–42. doi: 10.1016/j.ebiom.2018.01.025, PMID: 29398595 PMC5835570

[2] Ponticelli C Reggiani F Moroni G . Delayed graft function in kidney transplant: risk factors, consequences and prevention strategies. J Pers Med. (2022) 12:1557. doi: 10.3390/jpm12101557, PMID: 36294695 PMC9605016

[3] Strandberg G Öberg CM Blom AM Slivca O Berglund D Segelmark M . Prompt thrombo-inflammatory response to ischemia-reperfusion injury and kidney transplant outcomes. Kidney Int Rep. (2023) 8:2592–602. doi: 10.1016/j.ekir.2023.09.025, PMID: 38106604 PMC10719603

[4] Błogowski W Dołęgowska B Sałata D Budkowska M Domański L Starzyńska T . Clinical Analysis of Perioperative Complement Activity during Ischemia/Reperfusion Injury following Renal Transplantation. Clin J Am Soc Nephrol. (2012) 7:1843. doi: 10.2215/CJN.02200312, PMID: 22904122 PMC3488944

[5] Arias-Cabrales CE Riera M Pérez-Sáez MJ Gimeno J Benito D Redondo D . Activation of final complement components after kidney transplantation as a marker of delayed graft function severity. Clin Kidney J. (2020) 14:1190–6. doi: 10.1093/ckj/sfaa147, PMID: 33841865 PMC8023215

[6] Jackson AM Amato-Menker C Bettinotti M . Cell-free DNA diagnostics in transplantation utilizing next generation sequencing. Hum Immunol. (2021) 82:850–8. doi: 10.1016/j.humimm.2021.07.006, PMID: 34600770

[7] Oellerich M Sherwood K Keown P Schütz E Beck J Stegbauer J . Liquid biopsies: donor-derived cell-free DNA for the detection of kidney allograft injury. Nat Rev Nephrol. (2021) 17:591–603. doi: 10.1038/s41581-021-00428-0, PMID: 34031575

[8] Jansen MPB Emal D Teske GJD Dessing MC Florquin S Roelofs JJTH . Release of extracellular DNA influences renal ischemia reperfusion injury by platelet activation and formation of neutrophil extracellular traps. Kidney Int. (2017) 91:352–64. doi: 10.1016/j.kint.2016.08.006, PMID: 27692564

[9] van Zyl M Cramer E Sanders JSF Leuvenink HGD Lisman T van Rooy MJ . The role of neutrophil extracellular trap formation in kidney transplantation: Implications from donors to the recipient. Am J Transpl. (2024) 24:1547–57. doi: 10.1016/j.ajt.2024.04.018, PMID: 38719094

[10] Björk J Grubb A Sterner G Nyman U . Revised equations for estimating glomerular filtration rate based on the Lund-Malmö Study cohort. Scand J Clin Lab Invest. (2011) 71:232–9. doi: 10.3109/00365513.2011.557086, PMID: 21391777

[11] Leffler J Martin M Gullstrand B Tydén H Lood C Truedsson L . Neutrophil extracellular traps that are not degraded in systemic lupus erythematosus activate complement exacerbating the disease. J Immunol. (2012) 188:3522–31. doi: 10.4049/jimmunol.1102404, PMID: 22345666

[12] Cucchiari D Cuadrado-Payan E Gonzalez-Roca E Revuelta I Argudo M Ramirez-Bajo MJ . Early kinetics of donor-derived cell-free DNA after transplantation predicts renal graft recovery and long-term function. Nephrol Dial Transpl. (2023) 39:114–21. doi: 10.1093/ndt/gfad120, PMID: 37715343

[13] Kim K Moon H Lee YH Seo JW Kim YG Moon JY . Clinical relevance of cell-free mitochondrial DNA during the early postoperative period in kidney transplant recipients. Sci Rep. (2019) 9:18607. doi: 10.1038/s41598-019-54694-x, PMID: 31819080 PMC6901568

[14] Shen J Zhou Y Chen Y Li X Lei W Ge J . Dynamics of early post-operative plasma ddcfDNA levels in kidney transplantation: a single-center pilot study. Transpl Int. (2019) 32:184–92. doi: 10.1111/tri.13341, PMID: 30198148

[15] Gielis EM Beirnaert C Dendooven A Meysman P Laukens K De Schrijver J . Plasma donor-derived cell-free DNA kinetics after kidney transplantation using a single tube multiplex PCR assay. PLoS One. (2018) 13:e0208207. doi: 10.1371/journal.pone.0208207, PMID: 30521549 PMC6283554

[16] Lane R Nie J Kayler LK . Donor-derived cell-free DNA as a graft injury marker following kidney transplantation. Transplant Direct. (2022) 8:e1301. doi: 10.1097/TXD.0000000000001301, PMID: 35368988 PMC8966958

[17] Kusaka M Kawai A Takahara K Sasaki H Ito T Kenmochi T . Total cell-free DNA as a noninvasive biomarker of a delayed graft function after kidney transplantation from donors after cardiac death. Transplant Proc. (2023) 55:733–6. doi: 10.1016/j.transproceed.2023.03.008, PMID: 37031037

[18] Jansen MPB Pulskens WPC Uil M Claessen N Nieuwenhuizen G Standaar D . Urinary mitochondrial DNA associates with delayed graft function following renal transplantation. Nephrol Dial Transpl. (2020) 35:1320–7. doi: 10.1093/ndt/gfy372, PMID: 30590723

[19] Wei L Zhao Y Deng S Wu S Wang H Luo X . Graft-derived cell free DNA: used for assessment of early graft status and its implications for long-term kidney function. Front Physiol. (2024) 15:1440799. doi: 10.3389/fphys.2024.1440799, PMID: 39619094 PMC11604621

[20] Yang H Hou Y Liang T Lan Y He J Lu J . Quantification of postoperative graft-derived cell-free DNA to evaluate the risks of impaired allograft function at early stage of kidney transplantation. Transplant Proc. (2022) 54:2159–64. doi: 10.1016/j.transproceed.2022.08.030, PMID: 36369141

[21] Di W Ran Q Yang H Lu J Hou Y Wang X . Use of graft-derived cell-free DNA as a novel biomarker to predict allograft function after kidney transplantation. Int J Urol. (2021) 28:1019–25. doi: 10.1111/iju.14638, PMID: 34229363

[22] Nie W Su X Liu L Li J Fu Q Li X . Dynamics of donor-derived cell-free DNA at the early phase after pediatric kidney transplantation: A prospective cohort study. Front Med. (2022) 8:814517. doi: 10.3389/fmed.2021.814517, PMID: 35071284 PMC8777035

[23] Bloom RD Bromberg JS Poggio ED Bunnapradist S Langone AJ Sood P . Cell-free DNA and active rejection in kidney allografts. J Am Soc Nephrol. (2017) 28:2221. doi: 10.1681/ASN.2016091034, PMID: 28280140 PMC5491290

[24] Jordan SC Bunnapradist S Bromberg JS Langone AJ Hiller D Yee JP . Donor-derived cell-free DNA identifies antibody-mediated rejection in donor specific antibody positive kidney transplant recipients. Transplant Direct. (2018) 4:e379. doi: 10.1097/TXD.0000000000000821, PMID: 30234148 PMC6133406

[25] Xing Y Guo Q Wang C Shi H Zheng J Jia Y . Donor-derived cell-free DNA as a diagnostic marker for kidney-allograft rejection: A systematic review and meta-analysis. Biomol Biomed. (2024) 24:731–40. doi: 10.17305/bb.2024.10049, PMID: 38386614 PMC11293223

[26] Bromberg JS Bunnapradist S Samaniego-Picota M Anand S Stites E Gauthier P . Elevation of donor-derived cell-free DNA before biopsy-proven rejection in kidney transplant. Transplantation. (2024) 108:1994–2004. doi: 10.1097/TP.0000000000005007, PMID: 38595232 PMC11335081

[27] Bu L Gupta G Pai A Anand S Stites E Moinuddin I . Clinical outcomes from the Assessing Donor-derived cell-free DNA Monitoring Insights of kidney Allografts with Longitudinal surveillance (ADMIRAL) study. Kidney Int. (2022) 101:793–803. doi: 10.1016/j.kint.2021.11.034, PMID: 34953773

[28] Granata S Votrico V Spadaccino F Catalano V Netti GS Ranieri E . Oxidative stress and ischemia/reperfusion injury in kidney transplantation: focus on ferroptosis, mitophagy and new antioxidants. Antioxidants. (2022) 11:769. doi: 10.3390/antiox11040769, PMID: 35453454 PMC9024672

[29] Pefanis A Ierino FL Murphy JM Cowan PJ . Regulated necrosis in kidney ischemia-reperfusion injury. Kidney Int. (2019) 96:291–301. doi: 10.1016/j.kint.2019.02.009, PMID: 31005270

[30] Strandberg G- Raihle. C Nilsson B Öberg CM Blom AM Axman S . Early postreperfusion proteomics reveal divergent inflammatory responses in kidney transplantation with implications on outcomes. Transplantation. (2025). doi: 10.1097/TP.0000000000005561, PMID: 41171603 PMC12908640

[31] Trouw LA Nilsson SC Gonçalves I Landberg G Blom AM . C4b-binding protein binds to necrotic cells and DNA, limiting DNA release and inhibiting complement activation. J Exp Med. (2005) 201:1937–48. doi: 10.1084/jem.20050189, PMID: 15967823 PMC2212022

[32] Thålin C Aguilera K Hall NW Marunde MR Burg JM Rosell A . Quantification of citrullinated histones: Development of an improved assay to reliably quantify nucleosomal H3Cit in human plasma. J Thromb Haemost. (2020) 18:2732–43. doi: 10.1111/jth.15003, PMID: 32654410 PMC8722705

[33] Wang Y Li M Stadler S Correll S Li P Wang D . Histone hypercitrullination mediates chromatin decondensation and neutrophil extracellular trap formation. J Cell Biol. (2009) 184:205–13. doi: 10.1083/jcb.200806072, PMID: 19153223 PMC2654299

[34] Zhang F Li Y Wu J Zhang J Cao P Sun Z . The role of extracellular traps in ischemia reperfusion injury. Front Immunol. (2022) 13:1022380. doi: 10.3389/fimmu.2022.1022380, PMID: 36211432 PMC9533173

[35] Wu X You D Cui J Yang L Lin L Chen Y . Reduced neutrophil extracellular trap formation during ischemia reperfusion injury in C3 KO mice: C3 requirement for NETs release. Front Immunol. (2022) 13:781273. doi: 10.3389/fimmu.2022.781273, PMID: 35250972 PMC8889019

[36] Liu Y Xin Y Yuan M Liu Y Song Y Shen L . Sivelestat sodium protects against renal ischemia/reperfusion injury by reduction of NETs formation. Arch Biochem Biophys. (2025) 765:110318. doi: 10.1016/j.abb.2025.110318, PMID: 39863096

[37] Awad AS Rouse M Huang L Vergis AL Reutershan J Cathro HP . Compartmentalization of neutrophils in the kidney and lung following acute ischemic kidney injury. Kidney Int. (2009) 75:689–98. doi: 10.1038/ki.2008.648, PMID: 19129795 PMC2656389

[38] Stortz JA Hawkins RB Holden DC Raymond SL Wang Z Brakenridge SC . Cell-free nuclear, but not mitochondrial, DNA concentrations correlate with the early host inflammatory response after severe trauma. Sci Rep. (2019) 9:13648. doi: 10.1038/s41598-019-50044-z, PMID: 31541163 PMC6754448

[39] Magna M Pisetsky DS . The alarmin properties of DNA and DNA-associated nuclear proteins. Clin Ther. (2016) 38:1029–41. doi: 10.1016/j.clinthera.2016.02.029, PMID: 27021604

[40] Thurairajah K Briggs GD Balogh ZJ . The source of cell-free mitochondrial DNA in trauma and potential therapeutic strategies. Eur J Trauma Emerg Surg. (2018) 44:325–34. doi: 10.1007/s00068-018-0954-3, PMID: 29633007 PMC6002458

[41] Han F Wan S Sun Q Chen N Li H Zheng L . Donor plasma mitochondrial DNA is correlated with posttransplant renal allograft function. Transplantation. (2019) 103:2347. doi: 10.1097/TP.0000000000002598, PMID: 30747854

[42] Pollara J Edwards RW Lin L Bendersky VA Brennan TV . Circulating mitochondria in deceased organ donors are associated with immune activation and early allograft dysfunction. JCI Insight. (2018) 3:e121622. doi: 10.1172/jci.insight.121622, PMID: 30089724 PMC6129133

