# Supplementary material for: Nuclear cell-free DNA on the loose: an early warning signal of ischemia–reperfusion injury in kidney transplantation
Source: Front Immunol. 2026 Jan 7;16:1704152. doi: 10.3389/fimmu.2025.1704152 (PMC12819266; doi:10.3389/fimmu.2025.1704152)
Supplement: Supplementary file 1 [file DataSheet1.docx]

**Supplementary material**

**1.1 Supplementary Figure S1**

**Nuclear cfDNA release by DD kidney preservation method**

**
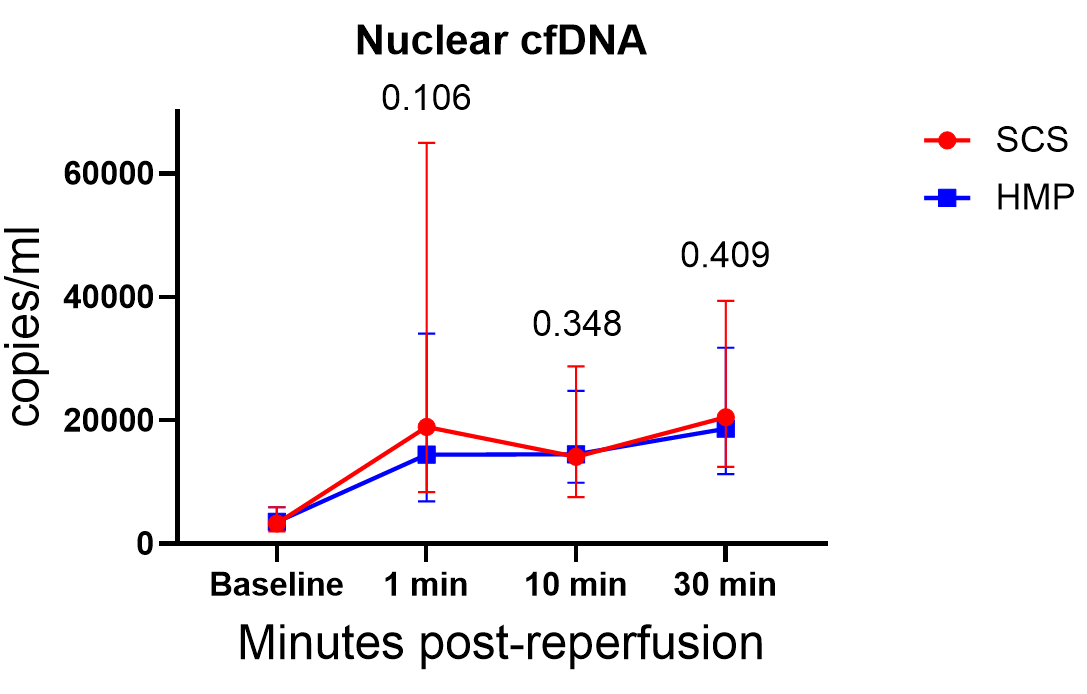
**

Supplementary Figure S1. Connected scatter plots depicting median (interquartile range) of nuclear cfDNA levels (copies/mL) by sampling time for recipients of DD kidneys, stratified by preservation method: non-oxygenated HMP (n=32) and SCS (n=54). To allow for visualization of baseline, plots present non-baseline-adjusted values for nuclear cfDNA. P values are presented for differences in baseline-subtracted levels between preservation methods by Mann–Whitney U tests. cfDNA = cell-free DNA, DD = deceased-donor, HMP = hypothermic machine perfusion, SCS = static cold storage.

**2.1 Supplementary Table S1**

**Spearman correlations of cfDNA and NETs markers**

| Spearman correlations | | | |
| --- | --- | --- | --- |
|  | | HNE-DNA | Citrullinated histone H3 |
| Nuclear cfDNA | 1  min | 0.212 (*P* = 0.121) | -0.132 (*P* = 0.335) |
|  | 10  min | **0.607 (*P < 0.001*)** | -0.140  (*P* = 0.314) |
|  | 30  min | **0.475 (*P < 0.001*)** | -0.181 (*P* = 0.191) |
|  | | | |
| Mitochondrial cfDNA | 1  min | **0.340 (*P* = 0.011)** | 0.095 (*P* = 0.491) |
|  | 10  min | 0.241 (*P* = 0.080) | 0.217 (*P* = 0.115) |
|  | 30  min | 0.056 (*P* = 0.687) | **0.285 (*P* = 0.037)** |

Supplementary Table S1. Spearman correlation coefficients of baseline-subtracted levels of HNE-DNA or citrullinated histone H3 and both nuclear and mitochondrial cfDNA levels at 1-, 10-, and 30-minutes post-reperfusion. Bold text indicates 2-tailed *P* < 0.05. cfDNA = cell-free DNA, HNE-DNA = human neutrophil elastase-DNA.

**2.2 Supplementary Table S2**

**Spearman correlations of NETs markers**

| Spearman correlations | | |
| --- | --- | --- |
|  | | HNE-DNA |
| Citrullinated histone H3 | 1  min | 0.210 (*P* = 0.124) |
|  | 10  min | -0.028 (*P* = 0.842) |
|  | 30  min | 0.051 (*P* = 0.712) |

Supplementary Table S2. Spearman correlation coefficients of baseline-subtracted levels HNE-DNA and citrullinated histone H3 at 1-, 10-, and 30-minutes post-reperfusion. HNE-DNA = human neutrophil elastase-DNA.

**2.3 Supplementary Table S3**

**Spearman correlations of cfDNA and sC5b-9**

| Spearman correlations | | | |
| --- | --- | --- | --- |
|  | | Nuclear cfDNA | Mitochondrial cfDNA |
| sC5b-9 | 1  min | **0.460 (*P* < 0.001)** | 0.243 (*P* = 0.066) |
|  | 10  min | **0.373 (*P* = 0.004)** | **0.286 (*P* = 0.031)** |
|  | 30  min | 0.160 (*P* = 0.24) | -0.011 (*P* = 0.937) |

Supplementary Table S3. Spearman correlation coefficients of baseline-subtracted levels of nuclear or mitochondrial cfDNA levels and sC5b-9 at 1-, 10-, and 30-minutes post-reperfusion. Bold text indicates 2-tailed *P* < 0.05. cfDNA = cell-free DNA, sC5b-9 = soluble sC5b-9.
